# Erythrocyte microRNA sequencing reveals differential expression in relapsing-remitting multiple sclerosis

**DOI:** 10.1186/s12920-018-0365-7

**Published:** 2018-05-21

**Authors:** Kira Groen, Vicki E. Maltby, Rodney A. Lea, Katherine A. Sanders, J. Lynn Fink, Rodney J. Scott, Lotti Tajouri, Jeannette Lechner-Scott

**Affiliations:** 1 School of Medicine and Public Health, University of Newcastle, Callaghan, NSW 2308 Australia; 2 Centre for Information Based Medicine, Level 3 West, Hunter Medical Research Institute, 1 Kookaburra Circuit, New Lambton Heights, NSW 2305 Australia; 3 Institute of Health and Biomedical Innovations, Genomics Research Centre, Queensland University of Technology, Kelvin Grove, QLD 4059 Australia; 4 Centre for Anatomical and Human Sciences, Hull York Medical School, Hull, HU6 7RX UK; 5 Diamantina Institute, University of Queensland, Woolloongabba, QLD 4102 Australia; 6 Division of Molecular Genetics, Pathology North, John Hunter Hospital, New Lambton Heights, NSW 2305 Australia; 7 School of Biomedical Sciences and Pharmacy, University of Newcastle, Callaghan, NSW 2308 Australia; 8 Faculty of Health Sciences and Medicine, Bond University, QLD, Robina, 4229 Australia; 9 Department of Neurology, John Hunter Hospital, New Lambton Heights, NSW 2305 Australia

**Keywords:** Erythrocytes, microRNA, Relapsing-remitting multiple sclerosis, Next-generation sequencing

## Abstract

**Background:**

There is a paucity of knowledge concerning erythrocytes in the aetiology of Multiple Sclerosis (MS) despite their potential to contribute to disease through impaired antioxidant capacity and altered haemorheological features. Several studies have identified an abundance of erythrocyte miRNAs and variable profiles associated with disease states, such as sickle cell disease and malaria. The aim of this study was to compare the erythrocyte miRNA profile of relapsing-remitting MS (RRMS) patients to healthy sex- and age-matched controls.

**Methods:**

Erythrocytes were purified by density-gradient centrifugation and RNA was extracted. Following library preparation, samples were run on a HiSeq4000 Illumina instrument (paired-end 100 bp sequencing). Sequenced erythrocyte miRNA profiles (9 patients and 9 controls) were analysed by DESeq2. Differentially expressed miRNAs were validated by RT-qPCR using miR-152-3p as an endogenous control and replicated in a larger cohort (20 patients and 18 controls). After logarithmic transformation, differential expression was determined by two-tailed unpaired t-tests. Logistic regression analysis was carried out and receiver operating characteristic (ROC) curves were generated to determine biomarker potential.

**Results:**

A total of 236 erythrocyte miRNAs were identified. Of twelve differentially expressed miRNAs in RRMS two showed increased expression (adj. *p* < 0.05). Only modest fold-changes were evident across differentially expressed miRNAs. RT-qPCR confirmed differential expression of miR-30b-5p (0.61 fold, *p* < 0.05) and miR-3200-3p (0.36 fold, *p* < 0.01) in RRMS compared to healthy controls. Relative expression of miR-3200-5p (0.66 fold, NS *p* = 0.096) also approached significance. MiR-3200-5p was positively correlated with cognition measured by audio-recorded cognitive screen (*r* = 0.60; *p* < 0.01). MiR-3200-3p showed greatest biomarker potential as a single miRNA (accuracy = 75.5%, *p* < 0.01, sensitivity = 72.7%, specificity = 84.0%). Combining miR-3200-3p, miR-3200-5p, and miR-30b-5p into a composite biomarker increased accuracy to 83.0% (p < 0.05), sensitivity to 77.3%, and specificity to 88.0%.

**Conclusions:**

This is the first study to report differences in erythrocyte miRNAs in RRMS. While the role of miRNAs in erythrocytes remains to be elucidated, differential expression of erythrocyte miRNAs may be exploited as biomarkers and their potential contribution to MS pathology and cognition should be further investigated.

**Electronic supplementary material:**

The online version of this article (10.1186/s12920-018-0365-7) contains supplementary material, which is available to authorized users.

## Background

Multiple Sclerosis (MS) is an autoimmune disease of the central nervous system (CNS) marked by lymphocytic infiltration, demyelination, and neurodegeneration. It affects approximately 2.5 million individuals worldwide. MS is a heterogeneous disease, which is divided into three disease courses with relapsing-remitting MS (RRMS) being the most common (around 85% of the patient population) [1, 2]. Its aetiology is assumed to be the interaction between environmental risk factors and genetic predisposition [3], however the exact cause and pathophysiology remain unclear.

MS is associated with activated peripheral and CNS-resident immune cells [2] and it remains to be determined whether erythrocytes play a role in its pathology. Erythrocytes are anucleate cells responsible primarily for respiratory gas transport [4, 5], yet are also thought to play a dynamic role in health and disease [6]. The potential involvement of erythrocytes in MS has been recently reviewed [7]. Briefly, altered haemorheological features of erythrocytes have been documented in MS and may be contributing to blood-brain barrier (BBB) disruption, a hallmark of MS pathology [2]. Furthermore, erythrocytes may contribute to increased levels of oxidative stress in MS through impaired antioxidant enzyme capacity [7]. In addition to MS pathology, disease-modifying therapies (DMTs) also appear to affect erythrocytes. For instance, natalizumab has been shown to result in previously undetected circulating erythrocyte precursors [8–10]. Mitoxantrone [11], fingolimod [12], and dimethyl fumarate [13] all have the potential to cause eryptosis, erythrocyte-specific apoptosis, and interferon-β has been shown to reduce red cell distribution width, possibly altering haemorheological features [14].

More recent studies have focused on the potential use of erythrocytes as MS biomarkers, using exogenous C-peptide binding to erythrocytes [15]. Biomarkers that can accurately reflect pathological and physiological processes are crucial in diseases as complex and heterogeneous as MS. Such biomarkers are needed for diagnosis and patient stratification, but also monitoring of treatment efficacy and disease progression [16]. Current MS diagnosis and monitoring relies on procedures such as lumbar punctures and magnetic resonance imaging (MRI) of the brain and spinal cord [2]. MicroRNA (miRNA) profiles are gaining increasing interest as MS biomarkers as they can reflect a range of ongoing pathological and physiological processes simultaneously [16, 17]. MiRNAs are small (~ 22 bp) non-coding RNA molecules that control gene expression at the post-transcriptional level [18]. Studies have shown that in peripheral blood mononuclear cell (PBMC) miRNAs can accurately differentiate between MS patients and healthy controls (HCs) [19, 20]; however, PBMCs only make up a very small percentage of whole blood elements [4]. The majority of a blood sample, erythrocytes and plasma, is discarded when assessing PBMCs. Additionally, some DMTs, such as fingolimod, are known to decrease circulating PBMC numbers [21]. Consequently, PBMC-derived miRNA profiles may not be the most suitable biomarker to monitor MS. Erythrocytes, which are abundant and can be quickly and cost-effectively purified [4], may lend themselves as a superior option.

Recent expression studies have identified an amplitude of miRNA transcripts in circulating erythrocytes [6, 22, 23]. Erythrocyte miRNA profiles were found to differ from leukocyte and reticulocyte profiles, yet largely reflect the miRNA expression of whole blood [6]. While the exact role of miRNAs in translationally inactive erythrocytes [24] is still unknown, they may be involved in intercellular communication through erythrocyte-derived extracellular vesicles (EVs) [25], or remnants of a functional erythrocyte precursor transcriptome [6, 24]. Despite uncertainty regarding the function of erythrocyte miRNAs, they have been found to differ in health and disease [6]. The use of erythrocyte-specific miRNA profiles is advantageous to whole blood miRNAs, as it eliminates variation that may arise from differences in whole blood cell composition. Erythrocytes have an average lifespan of 120 days in healthy individuals [4], but reduced lifespans have been reported in athletes [26] and some disease states [27]. Therefore, erythrocyte miRNAs may prove to present a relatively stable picture of miRNA expression, whereas translationally active cell miRNA profiles tend to only provide a snapshot of current miRNA expression, subjective to day-to-day variation [28]. However, this hypothesis demands further investigation.

Erythrocyte miRNAs may be exploited as biomarkers for MS patient stratification, diagnosis, and monitoring of treatment response and disease progression. Next-generation sequencing (NGS) technology has the potential to identify novel MS miRNA signatures in erythrocytes. The aim of this study was to characterise the erythrocyte miRNA profile of RRMS patients and compare it to HCs using NGS technologies.

## Methods

### Sample collection

Ethical approval was obtained from the Bond University Human Research Ethics Committee (RO-1382), the University of Newcastle Ethics Committee (H-505-0607), and the Hunter New England Health Ethics Committee (05/04/13/3.09). All participants gave written, informed consent prior to enrolment. Whole blood was collected into EDTA tubes from an initial cohort of 9 female RRMS patients and 9 female healthy controls (HCs). A further 20 female RRMS and 18 female HC samples were collected as part of the replication cohort. RRMS diagnosis was defined according to the McDonald criteria [29]. Participants who were pregnant, breastfeeding, or suffering from an autoimmune condition other than MS were excluded from the study. To minimise confounders associated with sex, only females were recruited for this pilot study. Additional patient information was obtained through MSBase, an observational database open to neurologists and health care teams [30]. Participant characteristics are summarised in Table 1.

**Table 1:** Participant characteristics by cohort (sequencing and replication) and group (control subjects and RRMS patients)

		Sequencing Cohort		Replication Cohort	
		Control Subjects (*n* = 9)	RRMS Patients (*n* = 9)	Control Subjects (*n* = 18)	RRMS Patients (*n* = 20)
Female		100%	100%	100%	100%
Caucasian		100%	90%	94%	100%
Age (years)		34.75 (±11.45)	42.44 (±9.66)	38.92 (± 9.89)	38.39 (±9.41)
Disease Duration (years)		N/A	15.11 (±11.87)	N/A	9.29 (±.5.52)
EDSS Score			2.00 (±1.32)		2.09 (±1.53)
Age at Onset (years)			27.94 (±8.48)		28.96 (± 8.79)
Number of Relapses			5.98 (±4.91)		6.35 (± 3.33)
Days since Last Relapse			1083.00 (±1271.81)		985 (±969.75)
Treatment	Off Treatment		2		0
	Dimethyl fumarate		2		0
	Fingolimod		3		10
	Natalizumab		2		10
Time on Treatment (years)			2.46 (±2.60)		3.57 (±2.20)
Number of Relapses on Treatment			0.56 (±0.73)		1.50 (±2.12)
Most recent ARCS^a^			78.33 (±26.76)		81.35 (±20.43)

Except for percentages and absolute numbers, all data is presented as mean (± SD). *RRMS* relapsing-remitting multiple sclerosis, *EDSS* extended disability status scale, *ARCS* audio-recorded cognitive screen, *SD* standard deviation of the mean. ^a^ARCS are only reported for patients who completed an ARCS within a year of sample collection (sequencing cohort *n* = 3; replication cohort *n* = 17)

### Erythrocyte purification

Erythrocytes were purified from 10 ml whole blood by density-gradient centrifugation. Lymphoprep (Stem Cell Technologies, Canada) density-gradient media was used according to manufacturer protocol. Plasma, PBMC layer, density-gradient media, and top erythrocyte layer were aspirated. The remaining erythrocyte pellet was washed twice with Hanks-balanced salt solution (HBSS) (GE Healthcare, United Kingdom).

### Purity assessment of erythrocytes

Purity of obtained erythrocytes was assessed by flow cytometry. One μl of erythrocyte pellet was stained with FITC-conjugated CD235a (Clone 2B7; erythrocytes and precursors) (BD Pharmingen, USA) and PE-conjugated CD71 (M-A712; reticulocytes) (BD Pharmingen, USA). All samples met a minimum purity cut-off of 95%. Samples were analysed on FACS Canto II (BD Biosciences, USA) using FACS Diva Software (BD Biosciences, USA).

### RNA extraction

Total RNA was extracted from 300 μl erythrocyte pellets with miRNeasy Kits (Qiagen, USA). Pellets were homogenized by vortexing samples for 1 min. Total RNA was quantified using the broad-range RNA Kit for the Qubit 2.0 (Life Technologies, USA). RNA integrity was determined with the RNA 6000 Nano kit on a 2100 Bioanalyzer (Agilent Technologies, USA).

### MiRNA sequencing and analysis

Library preparation and sequencing were performed by the Diamantina Institute, University of Queensland, Brisbane, Australia. Libraries were prepared with TruSeq Small RNA Library Preparation kits (Illumina, USA). Samples were individually barcoded and then sequenced in two multiplexed pools. Samples were run on the HiSeq4000 platform (Illumina, USA) using paired-end sequencing (read length: 100 bp; coverage 1 million reads/sample). Sequencing reads were de-multiplexed using CASAVA 1.8 software package (Illumina, USA) and adapter sequences were trimmed using Trim Galore! (https://www.bioinformatics.babraham.ac.uk/projects/trim_galore/). Reads were aligned against miRBase 21 [31] using STAR [32] and NGS results were analysed by DESeq2 [33]. Significance was adjusted for false discovery rate (FDR) using the Benjamini-Hochberg procedure.

### Reverse transcription quantitative polymerase chain reaction (RT-qPCR) – Validation and replication

Differentially expressed erythrocyte miRNAs flagged by NGS were confirmed in the initial NGS cohort and in a replication cohort of 20 RRMS patients and 18 HCs using TaqMan Advanced miRNA Assays (Assay IDs: hsa-let-7f-5p: 478578, hsa-miR-30b-5p: 478007, hsa-miR-32-5p: 478026, hsa-96-5p: 478215, hsa-181a-5p: 477857, hsa-miR-362-5p: 478059, hsa-598-3p: 478172, hsa-652-3p: 478189, hsa-miR-660-5p: 478192, hsa-miR-1294: 478693, hsa-miR-3200-3p: 478322, hsa-miR-3200-5p: 478021; Applied Biosystems, Thermo Fisher Scientific, USA). MiR-152-3p (hsa-miR-152-3p: 477921; Applied Biosystems, Thermo Fisher Scientific, USA) was used as an endogenous normalisation control based on a previous study investigating erythrocyte miRNAs by RT-qPCR [6]. The expression of miR-152-3p did not differ between RRMS patients and healthy controls in our sequencing cohort (Additional file 1: Table S1). Additionally, miR-152-3p expression was not altered during different stages of erythroid differentiation [34], it demonstrated the least variation across 40 different human tissue samples (Applied Biosystems, unpublished data, see reference [6]), and showed great stability in a hepatic study [34]. Relative expression was calculated using the 2^-deltaCT^ method. All RT-qPCR experiments were performed on a ViiA 7 (Applied Biosystems, USA) instrument. IBM SPSS Statistics 24 was used for statistical analysis. Since this was a relatively small discovery-based project we chose to set a relaxed significance threshold of 0.05 so as to reduce true positive rejection rate (Type II error). Replication of significant hits using an independent replication cohort was performed the reduce the false positive rate (Type I error). Following logarithmic transformation, relative expression determined by RT-qPCR was assessed by two-tailed unpaired Student’s t-tests or ANOVA, depending on the number of groups to be compared. Receiver operating characteristic (ROC) curves were generated to assess the diagnostic value of confirmed miRNAs and accuracy was determined by area under the curve (AUC). Using relative expression cut-off values that resulted in greatest sensitivity and specificity for confirmed miRNAs, logistic regression analysis was performed in IBM SPSS Statistics 24 to determine the value of composite biomarkers using multiple confirmed miRNAs. Pearson correlation coefficients between confirmed miRNA and recorded disease outcome measures (Table 1) were calculated.

### MiRNA target prediction

MiRSystem was used to predict target genes of differentially expressed miRNAs as it integrates seven prediction algorithms and includes experimental validation of miRNA-mRNA interactions [35].

## Results

NGS was used to determine erythrocyte miRNA profiles of 9 RRMS patients and 9 HCs. RT-qPCR was then utilised to confirm differential expression of erythrocyte miRNAs revealed by NGS in the sequencing cohort and a more uniform replication cohort of 20 RRMS patients and 18 HC samples. Participant characteristics of the sequencing and replication cohort are shown in Table 1.

### Total RNA

Total erythrocyte RNA was extracted from 300 μl erythrocyte pellets (mean purity determined by flow cytometry was 99%, with < 1% leukocytes and platelets; further detail in Additional file 2: Table S2). Total erythrocyte RNA content in 10 ml whole blood was calculated by multiplying RNA yields from one aliquot by the number of aliquots obtained. Mean erythrocyte RNA yield obtained from 10 ml whole blood of MS patients (*n* = 29) was 20.12 μg (± 17.02 μg), while the mean erythrocyte RNA content of HCs (*n* = 27) was 12.44 μg (± 3.85 μg)/10 ml whole blood (*p* < 0.05). RNA yield did not correlated with bench time (time that passed between sample collection and processing) (MS patients: *r* = 0.145, *p* = 0.553; HC: *r* = 0.169, *p* = 0.502). Patients treated with natalizumab (32.35 μg/10 ml whole blood) had significantly greater RNA yields than patients on fingolimod (15.52 μg/10 ml whole blood, *p* < 0.01) and HCs (12.44 μg/10 ml whole blood, *p* < 0.001) (Additional file 3: Figure S1).

### Next-generation sequencing results

**Fig. 1:**
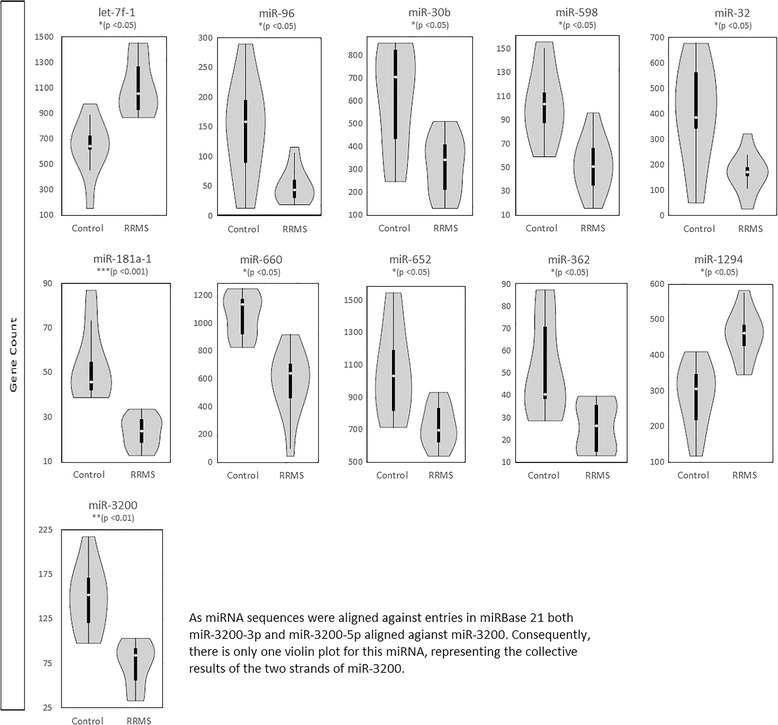
Violin plots of differentially expressed erythrocyte microRNAs identified by next-generation sequencing. The violin plots show normalised gene counts of erythrocyte miRNAs that were found to be differentially expressed in RRMS patients (*n* = 9) compared to HCs (*n* = 9). Sequences were aligned against miRBase 21 and differential expression was computed with DESeq2

Following TruSeq Small RNA library preparation (Illumina, USA), erythrocyte samples were sequenced by paired-end 100 bp sequencing on a HiSeq4000 (Illumina, USA) instrument, aiming for 1 million reads per sample. NGS revealed 236 known miRNAs across patient and control erythrocytes. Normalised gene counts ranged from 1 to > 21,800 (Additional file 1: Table S1). Twelve erythrocyte miRNAs were found to be differentially expressed in RRMS patients, two of which, miR-1294 (1.55-fold, FDR adj. *p* < 0.05) and let-7f-5p (1.73-fold, adj. p < 0.05), showed increased expression in RRMS compared to HCs. The remaining ten miRNAs, miR-181a-5p (0.45 fold, adj. *p* < 0.0001), miR-96-5p (0.43 fold, adj. *p* < 0.05), miR-32-5p (0.49 fold, adj. *p* < 0.05), miR-598-3p (0.53 fold, adj. p < 0.05), miR-362-5p (0.59 fold, adj. *p* < 0.05), miR-30b-5p (0.60 fold, adj. p < 0.05), miR-660-5p (0.64 fold, adj. *p* < 0.05), miR-652-3p (0.69 fold, adj. p < 0.05), and miR-3200-3p and -5p (0.85 fold, adj. *p* < 0.01), showed decreased expression in RRMS compared to HCs (Fig. 1). Four of the differentially expressed miRNAs (miR-181a-5p, miR-362-5p, miR-598-3p, and miR-96-5p) were very lowly expressed (< 100 reads) and only let-7f-5p, miR-660-5p, and miR-652-3p reached more than 800 reads per sample (Additional file 1: Table S1). No obvious clustering of patients and controls was evident when looking at global differences in miRNA expression.

### Reverse transcription quantitative polymerase chain reaction results – Validation and replication

To validate NGS results, differentially expressed erythrocyte miRNAs were assessed by RT-qPCR in the original sequencing cohort and a replication cohort.

**Fig. 2:**
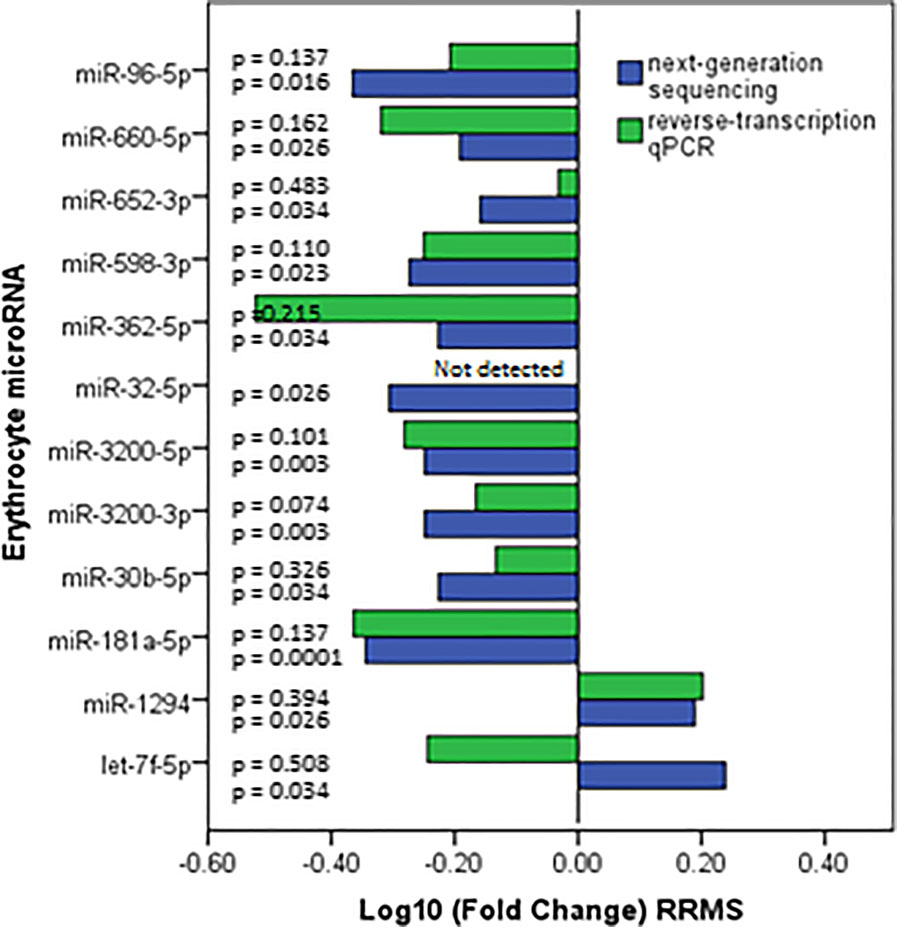
Log10(fold changes) of differentially expressed erythrocyte microRNAs in relapsing-remitting Multiple Sclerosis. Log10(fold changes) of differentially expressed erythrocyte miRNAs in relapsing-remitting Multiple Sclerosis (RRMS) patients compared to healthy controls (HC) by next-generation sequencing (9 RRMS patients and 9 HC) (blue) and RT-qPCR (9 RRMS patients and 5 HC) (green)

Samples that did not meet our quality control cut-off were removed from the dataset. Differential expression trends in the discovery cohort (9 RRMS patients and 5 HCs) were replicated for all miRNAs with the exception of let-7f-5p (Fig. 2). While the significance threshold (*p* < 0.05) was not reached, this may be a result of insufficient power to detect a significant change. With the aim of assessing whether differential erythrocyte miRNA expression was driven by DMTs as opposed to disease, the original sequencing cohort was segregated by DMT. No formal analysis was carried out due to lack of power, yet visual representation shows that with the exception of miR-3200-3p, miR-3200-5p, and miR-652-3p, off treatment RRMS patients’ erythrocyte miRNA expression resembles that of patients on DMT (Additional file 4: Figure S2).

**Fig. 3:**
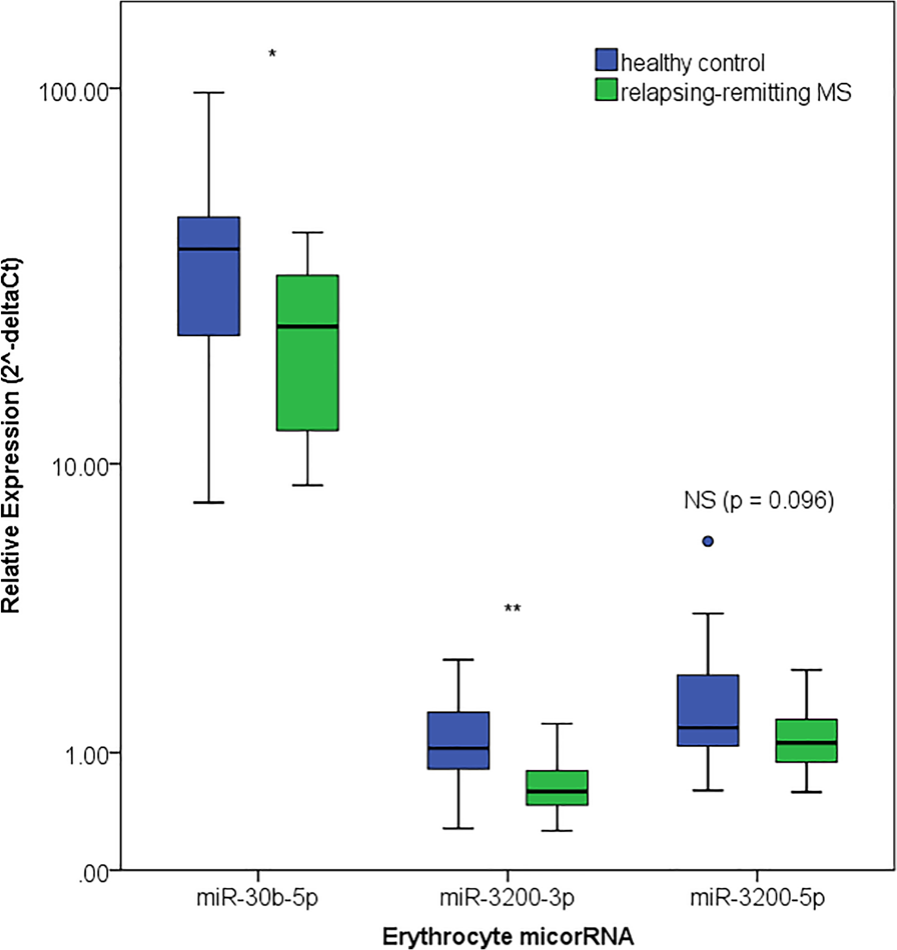
Tukey boxplot of differentially expressed erythrocyte microRNAs confirmed by reverse transcription polymerase chain reaction. Tukey boxplot of relative expression (2^-deltaCT^) (y-axis on a log scale) of erythrocyte microRNAs in relapsing-remitting MS patients (*n* = 20; green) and healthy controls (*n* = 18; blue). The blue dot represents an outlier defined as deviating ≥1.5 fold from the upper/lower quartile. * *p* < 0.05; ** *p* < 0.01; NS – not significant (*p* > 0.05)

RT-qPCR experiments were replicated in a larger, more uniform cohort of 20 RRMS patients and 18 HCs. Decreased expression of miR-30b-5p (0.61 fold, *p* < 0.05) and miR-3200-3p (0.36 fold, *p* < 0.01) was confirmed. Decreased expression of miR-3200-5p (0.66 fold, NS *p* = 0.096) approached significant threshold and was hence included in further analysis (Fig. 3). To assess the biomarker potential of the confirmed miRNAs, ROC curve analysis was performed for miRNAs that showed differential expression. ROC curves graphically illustrate the diagnostic potential of a binary outcome, in this case RRMS or HC, at different thresholds. This allows the determination of true and false positives for each of the thresholds, and specificity and sensitivity to be calculated.

**Fig. 4:**
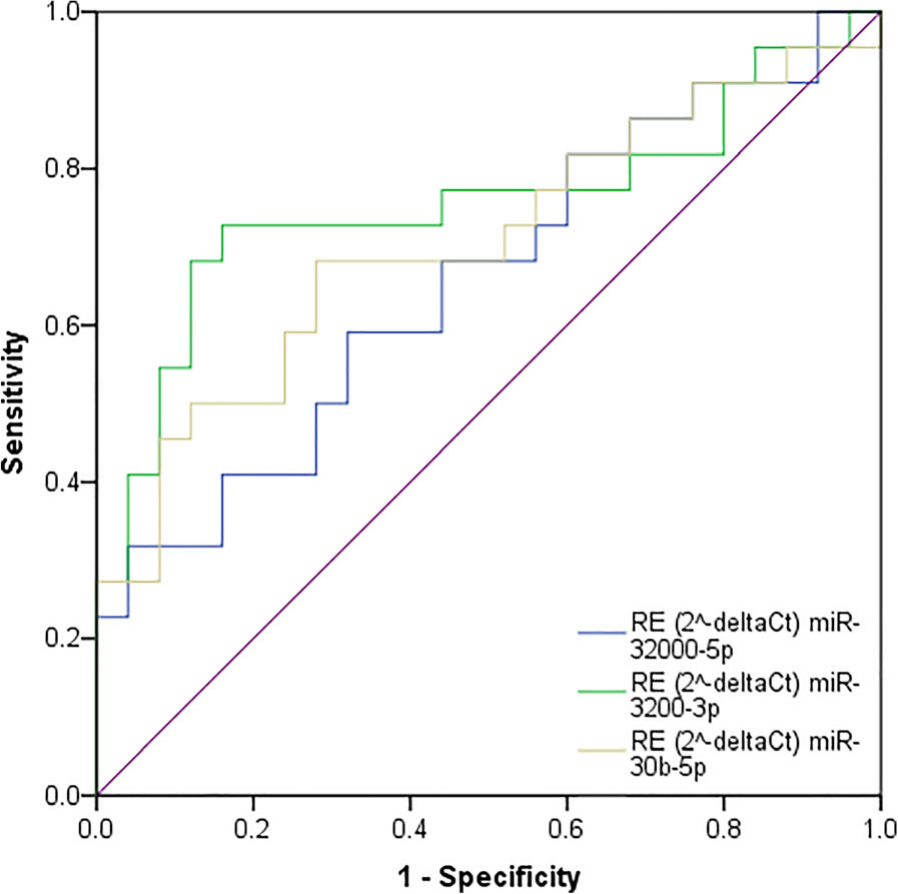
Receiver operating characteristic (ROC) curves for confirmed erythrocyte microRNAs. ROC curves showing sensitivity and 1-specificity at different thresholds for miR-3200-3p (green), miR-3200-5p (blue), and miR-30b-5p (yellow)

Cut-off values that maximised both specificity and sensitivity were chosen. MiR-3200-3p showed the greatest biomarker potential being able to distinguish between RRMS patients and HCs with 75.5% accuracy (*p* < 0.01; relative expression cut-off: 0.81), 72.7% sensitivity, and 84.0% specificity. MiR-30b-5p and miR-3200-5p were able to differentiate between RRMS and HCs with 70.5% (p < 0.05) and 65.8% (*p* = 0.064) accuracy, 68.2% and 59.1% sensitivity, and 72.0% and 68.0% specificity respectively (Fig. 4; Table 2). Combining relative expression (using cut-off values in Table 2) of the three confirmed miRNAs (miR-30b-5p, miR-3200-3p, and miR-3200-5p) in a binary logistic regression with MS or HC being the dichotomous outcome, cases and controls could be assigned to their respective category with overall accuracy of 83.0%, 77.3% sensitivity, and 88.0% specificity (Table 3).

**Table 2:** Receiver operating characteristic curve (ROC) results for the three confirmed erythrocyte microRNAs

MicroRNA	RE Cut-Off	Sensitivity (%)	Specificity (%)	Accuracy (%)	*p*-value
miR-30b-5p	34.40	68.2	72.0	70.5	0.016
miR-3200-3p	0.81	72.7	84.0	75.5	0.003
miR-3200-5p	1.20	59.1	68.0	65.8	0.064

*RE* relative expression (2^-deltaCT^)

**Table 3:** Classification of relapsing-remitting Multiple Sclerosis patients and healthy controls based on three erythrocyte miRNAs

		Predicted		Percentage Correct (%)
		RRMS	HC	
Observed	RRMS	22	3	88.0
	HC	5	17	77.3
Overall percentage				83.0

*RRMS* relapsing-remitting Multiple Sclerosis, *HC* healthy control

**Fig. 5:**
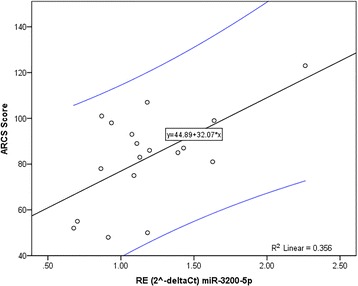
Linear regression for RE of miR-3200-5p and patients’ ARCS score. Relative expression (RE) of miR-3200-5p was positively correlated (correlation coefficient 0.597; p < 0.01) with patients’ audio-recorded cognitive screen (ARCS) score. Equations for the linear regression model (black line) and 95% confidence intervals (blue lines) are shown

To determined clinical impact of differentially expressed erythrocyte miRNAs Pearson correlation coefficients between confirmed miRNAs and recorded disease outcome measures (Table 1) were calculated. Relative expression of miR-3200-5p was positively correlated with patients most recent (within a year of sample collection) cognitive assessment measured by audio-recorded cognitive screen (ARCS) [36] (Pearson correlation coefficient: 0.597; p < 0.01) (Fig. 5), as well as some of the ARCS’ subdomains (Table 4). No further correlations between confirmed miRNAs and disease outcome measures, age, or bench time (elapsed time between blood collection and processing) were identified.

**Table 4:** Pearson correlation coefficients between miR-3200-5p and ARCS scores and subdomains

	Pearson Correlation Coefficient	*p*-value
Total ARCS	**0.597**	**0.009**
Quick ARCS	**0.592**	**0.010**
Memory Domain	0.427	0.077
Fluency Domain	**0.499**	**0.035**
Visuospatial Domain	0.314	0.205
Language Domain	0.282	0.256
Attention Domain	0.391	0.108
Speed of Writing	**0.634**	**0.005**

*ARCS* audio-recorded cognitive screen; significant correlations are highlighted in bold

There were no significant differences in miRNA levels between patients on natalizumab (*n* = 12) and fingolimod (*n* = 13) across the 12 assessed erythrocyte miRNAs.

### MiRNA target prediction

Given that fold changes were only modest for all differentially expressed miRNAs (Fig. 2), it is unlikely that a single miRNA is significantly affecting target gene expression. To this end, it was reasoned that several miRNAs may work in concert to target a few specific messenger RNAs (mRNAs). MiRSystem [35] was used to identify genes targeted by several of the 12 differentially expressed miRNAs identified by NGS. MiRSystem identified several genes targeted by four of the miRNAs flagged by NGS and one gene, *MIER 3* (mesoderm induction early response 1, family member 3), was targeted by five of the miRNAs identified as differentially expressed by NGS (Table 5).

**Table 5:** MicroRNA target prediction results

Target Gene	Gene Description	Observed microRNA
*MIER 3*	mesoderm induction early response 1, family member 3	miR-181a-5p, miR-30b-5p, miR-32-5p, miR-362-5p, miR-660-5p
*BCL2L11*	BCL2-like 11 (apoptosis facilitator)	miR-181a-5p, miR-30b-5p, miR-32-5p, miR-362-5p
*SEC24A*	SEC24, family member A	miR-181a-5p, miR-30b-5p, miR-32-5p, miR-660-5p
*SCN3A*	sodium channel, voltage-gated type III, alpha subunit	miR-30b-5p, miR-32-5p, miR-362-5p, miR-660-5p
*BCL11A*	B-cell CLL/lymphoma 11A (zinc finger protein)	miR-181a-5p, miR-30b-5p, miR-32-5p, miR-362-5p
*YTHDF3*	YTH domain family, member 3	miR-181a-5p, miR-30b-5p, miR-362-5p, miR-660-5p
*NFAT5*	nuclear factor of activated T-cells 5, tonicity response	miR-181a-5p, miR-30b-5p, miR-32-5p, miR-660-5p
*CPEB4*	cytoplasmic polyadenylation element binding protein 4	miR-181a-5p, miR-30b-5p, miR-32-5p, miR-660-5p
*LIN28*		miR-181a-5p, miR-30b-5p, miR-32-5p, miR-598-3p
*CNTN4*	contactin 4	miR-181a-5p, miR-30b-5p, miR-32-5p, miR-362-5p

## Discussion

This is the first study to compare the erythrocyte miRNA profile of RRMS patients to HCs. Differentially expressed erythrocyte miRNAs were identified in both the sequencing and replication cohort. Three miRNAs (miR-30b-5p, miR-3200-3p and miR-3200-5p) were found to show decreased expression in RRMS erythrocytes compared to HC erythrocytes and the combination of these miRNAs (miR-30b-5p, miR-3200-3p and miR-3200-5p) into a composite biomarker, was able to differentiate between RRMS patients and healthy controls with 77.3% sensitivity, 88.0% specificity, overall accuracy of 83.0%. Additionally, miR-3200-5p showed moderate correlation with patients’ cognitive function, determined by ARCS (correlation coefficient: 0.60; *p* < 0.01).

Current MS diagnosis is based on the 2013 revisions to the McDonald criteria and clinical evidence is a crucial component of this diagnosis [29]. Diagnosis and monitoring of MS is underpinned by demyelinating lesions on MRI and supported by positive oligoclonal bands in patients’ cerebrospinal fluid (CSF), requiring an invasive lumbar puncture. There is no reliable blood test that may guide diagnosis, monitoring, and selection of treatment options [1, 2]. Differential expression of miRNAs in erythrocytes, which can be easily obtained as part of other routine blood tests, indicates potential as supportive biomarkers for MS diagnosis and monitoring, reducing cost and patient discomfort associated with current paraclinical investigations. Erythrocytes are abundant in whole blood and thought to be translationally inactive; therefore, they lend themselves as stable biomarkers and should be further investigated. In order to establish the true diagnostic potential of erythrocyte miRNAs, their expression in MS needs to be compared to other differential diagnoses, this should be addressed by future studies. Differential diagnoses for MS include vascular diseases, such as systemic vasculitis, conditions of the brain and spinal cord, such as cerebellar ataxias, and tumours and structural lesions in the CNS [3]. Thus far, erythrocyte miRNAs have not been studied in diseases that make up the differential diagnoses for MS. Nonetheless, a study investigating whole blood miRNA profiles in CIS/RRMS patients and patients with neuromyelitis optica spectrum disorders (NMOSD) found differential expression of miR-30b in their discovery cohort (20 CIS/RRMS patients and 20 NMOSD patients) and replication cohort (19 RRMS/CIS patients and 18 NMOSD patients) [37]. As miR-30b was also found to be differentially expressed in this study and whole blood miRNAs have been found to reflect erythrocyte miRNAs [6], the aforementioned finding [37] underpins the potential erythrocyte miRNAs have as diagnostic biomarkers for MS. Studies have also compared whole blood miRNA profiles between stroke patients and healthy controls, highlighting differential expression patterns [38] and focusing on let-7e-5p expression [39]. With the differential expression patterns between stroke patients and healthy controls [38, 39] differing from the differential erythrocyte miRNA expression between healthy controls and RRMS patients identified by this study, one may argue that miRNAs can differentiate between stroke and RRMS patients, however this needs to be confirmed through further investigation.

NGS of erythrocyte miRNAs of female RRMS patients and HCs revealed 236 known miRNAs across RRMS and HC samples. Several of the miRNAs flagged by NGS, including the confirmed miR-3200-3p, have also been identified in Alzheimer’s Disease (AD). Satoh et al. identified decreased expression of let-7f-5p, miR-660-5p, miR-1294, and miR-3200-3p, as well as several others [40]. While not all trends match those in RRMS (Fig. 2), involvement of the same miRNAs in two distinct neurological diseases suggests their overall importance for the CNS. Satoh et al. used datasets from whole blood samples [40], not isolated erythrocytes, which raises the possibility of lymphocyte contamination. However, a previous study has shown that whole blood miRNAs largely reflect erythrocyte miRNAs [6], allowing for comparison between the two studies. Thus far, isolated erythrocyte miRNAs have not been studied in diseases other than malaria [41] and sickle cell disease [6].

One of the major symptoms of MS is cognitive impairment, which may develop in the absence of clinical relapse [2, 3]. Appropriate management of cognitive impairment is only possible if it can be detected early in the affected population [42]. Conventional neuropsychological assessment, the gold standard for detecting changes in cognition, is time-consuming, requires a trained psychologist, and is not feasible for routine clinical practice. Consequently, other cognitive screening instruments, such as the ARCS, have been developed; nonetheless, the ARCS still requires patients to spend 35 min in a quiet room [36]. No blood-borne biomarker for cognitive impairment has been implemented in clinical practice [43]. Differential expression of miR-3200-5p was correlated with patients’ most recent ARCS score (correlation coefficient: 0.597, *p* < 0.01), indicating that this miRNA may serve as a biomarker for cognitive function, reflecting global neuronal loss. The strongest correlation was observed between the subdomain speed of writing and the miRNA (correlation coefficient: 0.634, p < 0.01). Speed of writing requires patients to write out the word “table” as many times as possible in 30 s, reflecting information processing and fine motor skills [36]. The idea that circulating miRNAs may reflect cognitive impairment is not new: two small studies looked at mild cognitive impairment in the elderly and identified some miRNAs that correlated with mild cognitive impairment with high sensitivity and specificity [42, 43]. Cognitive decline is an early sign of MS and may reflect CNS damage more accurately than EDSS scores [44, 45]. Early detection for timely treatment and management are key to improve outcomes in MS, yet current cognitive screens are time and labour intensive and can be distressing for patients. The correlation between miR-3200-5p and patients’ ARCS score indicates this miRNA’s potential to be developed into a biomarker for cognitive impairment. Longitudinal assessment and validation in a larger cohort are necessary to confirm this hypothesis.

While a range of targets were predicted by miRSystem [35], fold changes in differentially expressed miRNAs were only minor, reducing the likelihood that a single miRNA is affecting gene expression. Nonetheless, several of the differentially expressed miRNAs were found to target the same genes, potentially amplifying dysregulation [18]. None of the predicted targets play an established role in mature erythrocytes and *MIER3* is not known to play a role in MS. The role of miRNAs in translationally inactive mature erythrocytes remains to be elucidated [24]. It has been suggested that erythrocyte miRNAs are remnants from earlier stages of erythrocyte development, where they played crucial roles in cell differentiation and maturation [6, 22]. Notwithstanding, erythrocyte miRNAs may play a more active role, functioning as intercellular communicators through erythrocyte-derived EVs. EVs are small, membrane-bound vesicles, containing proteins, nucleic acids, and lipids, which can be derived from a variety of cells, including erythrocytes [25].

None of the differentially expressed erythrocyte miRNAs were found to be highly abundant in erythrocytes (some of the most abundant miRNAs were miR-25, miR-144, miR-451, miR-182, and members of the let-7 family; Additional file 1: Table S1) and the reason for the observed differential expression remains to be clarified. Potential angles for investigation include stabilisation of certain miRNAs through associations with protein complexes, such as Argonaute proteins [22] and other non-coding RNAs [18], as well as targeted packaging and loss of certain miRNAs through EVs [25].

Recruited patients were on various DMTs (Table 1), some of which are known to alter erythrocyte phenotypes [8–13]. To account for the treatment effects, patients were recruited on a range of therapies, with the intent of identifying miRNA signatures that were disease- rather than treatment-specific. While lack of power did not allow for formal comparisons between RRMS patients on DMTs and untreated RRMS patients, visual comparison indicated that expression of miR-3200-3p, miR-3200-5p, and miR-652-3p might differ between these groups (Additional file 4: Figure S2). These differences and the effect of DMTs on erythrocyte miRNA expression needs to be further investigated and some of the findings of this study may be specific to MS patients on DMTs. Differences between DMTs were also assessed. Differential expression between natalizumab and fingolimod treated patients was evident for let-7f-5p, which showed increased expression in patients on fingolimod compared to patients on natalizumab (data not shown). While not statistically significant, this difference may explain why the trend of differential expression could not be replicated by RT-qPCR for let-7f-5p (Fig. 2). Neither of the treatment groups showed differential let-7f-5p expression compared to healthy controls (data not shown). Members of the let-7 family have been reported in other erythrocyte miRNA studies [6, 22, 23], where they are thought to be involved in erythropoiesis [46], and have also been shown to be less expressed in MS patients [47]. Cox et al. [47] used PAXGene (Qiagen, Germany) technology to analyse whole blood miRNA expression. Lack of similarity between identified miRNAs, other than let-7f, by Cox et al. and in this study, may reflect differences in cell make up, with PAXGene technology focussing mostly on leukocytes. Nevertheless, the reason for the difference in miRNA expression between treatments remains unknown and warrants further investigation.

Total RNA obtained from 10 ml whole blood varied between 9.40 and 35.35 μg (Additional file 1: Figure S1). Both reticulocytes and nucleated cells have been shown to harbour greater amounts of total RNA than erythrocytes [6]. Increased RNA yields from erythrocytes of patients treated with natalizumab may reflect increased levels of circulating erythrocyte precursors in these patients [8–10].

While low power to detect small differences in miRNA species and the recruitment of patients on different DMTs, some of which are known to alter erythrocytes [8–13], should be addressed by future studies, these preliminary results indicate that erythrocyte miRNAs should be incorporated into the growing list of MS biomarkers. Future investigations should aim to recruit larger numbers of treatment-naïve patients (including male MS patients), assess intra-individual variability, and evaluate specificity of observed differential expression to MS. In addition to healthy controls, future studies should aim to recruit pathological controls suffering from other systemic inflammatory, neurodegenerative, and autoimmune diseases.

The potential importance of erythrocyte pellets to MS and other diseases is starting to be recognized: Erythrocyte/granulocyte pellets are already being stored as part of the UK ME/CFS Biobank [48] and future investigations into erythrocytes and MS, or other autoimmune diseases, may provide a novel avenue for immunoregulatory prophylaxis and treatment options [49].

## Conclusions

This is the first study to explore erythrocyte miRNAs in RRMS. We find evidence to suggest that erythrocyte miRNAs, particularly miR-30b-5p, miR-3200-3p and miR-3200-5p, may be exploited as novel MS biomarkers and miR-3200-5p may be developed into a biomarker for cognitive decline. Further investigations are warranted to substantiate these findings as erythrocytes can be easily and cost-effectively purified and novel biomarkers are required to aid diagnosis and stratification of MS patients.

## Additional files

Additional file 1: **Table S1.** Next-generation sequencing results of erythrocyte microRNAs for 9 healthy controls and 9 relapsing-remitting Multiple Sclerosis patients. (XLSX 27 kb)

Additional file 2: **Table S2.** Erythrocyte purity determined by flow cytometry (*n* = 10). Data is shown as mean percent positive events out of 20,000 events. (XLSX 12 kb)

Additional file 3: **Figure S1.** Total erythrocyte RNA extracted from 10 ml whole blood by disease-modifying therapy. Mean total erythrocyte RNA yields from 10 ml whole blood for 2 patients off treatment, 2 on dimethyl fumarate, 13 on fingolimod, 12 on natalizumab and 27 healthy controls. Error bars represent standard deviation (SD). ** *p* < 0.01; *** *p* < 0.001. (PNG 41 kb)

Additional file 4 **Figure S2.** Tukey boxplot of relative expression (2^-deltaCt^) of differentially expressed erythrocyte microRNAs in the sequencing cohort by disease-modifying therapy. Relative expression (2^-deltaCt^) (y-axis on a logarithmic scale) of differentially expressed erythrocyte miRNAs (x-axis) by disease-modifying therapy (fingolimod: *n* = 3; natalizumab: *n* = 2; dimethyl fumarate: *n* = 2; off treatment: *n* = 2) and including healthy controls (*n* = 5). The dots represent outliers defined as deviating ≥1.5 fold from the upper/lower quartile. (PNG 29 kb)

## Abbreviations

AD: Alzheimer’s disease
ARCS: Audio-recorded cognitive screen
AUC: Area under the curve
BBB: Blood-brain barrier
CNS: Central nervous system
CSF: Cerebrospinal fluid
DMT: Disease-modifying therapy
EDSS: Extended disability status scale
EV: Extracellular vesicle
FDR: False discovery rate
HBSS: Hanks’ balanced salt solution
HC: Healthy control
miRNA: microRNA
MRI: Magnetic resonance imaging
mRNA: messenger RNA
MS: Multiple Sclerosis
NGS: Next-generation sequencing
NMOSD: Neuromyelitis optica spectrum disorder
PBMC: Peripheral blood mononuclear cell
RE: Relative expression
ROC: Receiver operating characteristic
RRMS: Relapsing-remitting Multiple Sclerosis
RT-qPCR: Reverse transcription quantitative polymerase chain reaction
SD: Standard deviation of the mean
