# Supplementary figures and images for: Erythrocyte microRNA sequencing reveals differential expression in relapsing-remitting multiple sclerosis
Source: BMC Med Genomics. 2018 May 21;11:48. doi: 10.1186/s12920-018-0365-7 (PMC5963124; doi:10.1186/s12920-018-0365-7)

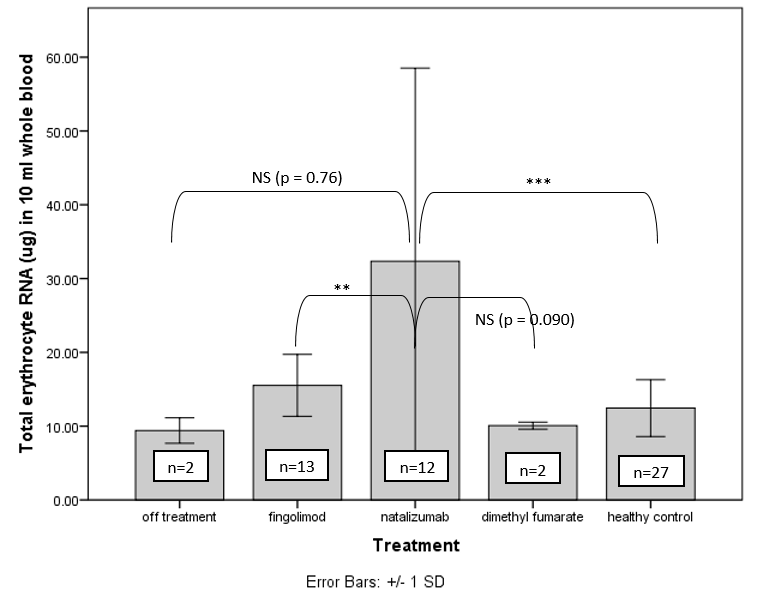

Supplement: Supplementary file 3 — Figure S1. Total erythrocyte RNA extracted from 10 ml whole blood by disease-modifying therapy. Mean total erythrocyte RNA yields from 10 ml whole blood for 2 patients off treatment, 2 on dimethyl fumarate, 13 on fingolimod, 12 on natalizumab and 27 healthy controls. Error bars represent standard deviation (SD). ** p < 0.01; *** p < 0.001. (PNG 41 kb) [file 12920_2018_365_MOESM3_ESM.png]

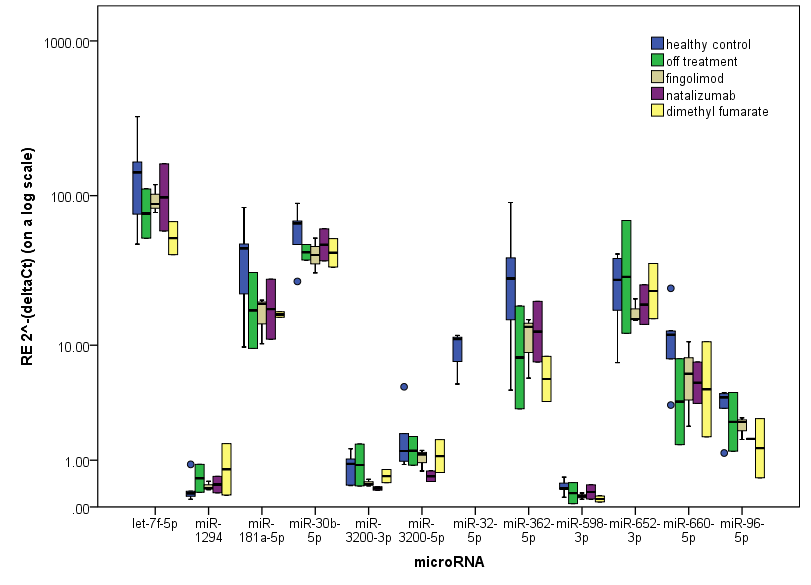

Supplement: Supplementary file 4 — Figure S2. Tukey boxplot of relative expression (2-deltaCt) of differentially expressed erythrocyte microRNAs in the sequencing cohort by disease-modifying therapy. Relative expression (2-deltaCt) (y-axis on a logarithmic scale) of differentially expressed erythrocyte miRNAs (x-axis) by disease-modifying therapy (fingolimod: n = 3; natalizumab: n = 2; dimethyl fumarate: n = 2; off treatment: n = 2) and including healthy controls (n = 5). The dots represent outliers defined as deviating ≥1.5 fold from the upper/lower quartile. (PNG 29 kb) [file 12920_2018_365_MOESM4_ESM.png]
